# A Scoping Review of Intervention Implementation for Self-Care Skills among Children with Developmental Delay

**DOI:** 10.21315/mjms2024.31.4.6

**Published:** 2024-08-27

**Authors:** Noor Amiera Alias, Masne Kadar, Farahiyah Wan Yunus, Katie Cremin

**Affiliations:** 1 Centre for Rehabilitation and Special Needs Studies, Occupational Therapy Programme, Faculty of Health Sciences, Universiti Kebangsaan Malaysia, Kuala Lumpur, Malaysia; 2 Centre of Occupational Therapy Studies, Faculty of Health Sciences, Universiti Teknologi MARA, Selangor, Malaysia; 3 Discipline of Occupational Therapy, Trinity College Dublin, Trinity Centre for Health Sciences, St James Hospital, Dublin 8, Ireland

**Keywords:** self-care skills, children, developmental delay, intervention, occupational therapy

## Abstract

Difficulties with self-care skills are frequently observed in children with developmental delays. Given the scarcity of robust evidence backing self-care interventions for this group, this scoping review is designed to aggregate existing literature on the implementation of such interventions. Therefore, this scoping review aims to collate literature on the nature of self-care intervention implementation to increase understanding of the current practice and inform future research directions. This scoping review endeavoured to explore the body of literature concerning the existence of self-care interventions and their implementation strategies in children who have developmental delays. Relevant studies were identified by searching through the following databases: Web of Science (W0S), Scopus, ASEAN Citation Index (ACI), CINAHL EBSCO and PubMed. Six types of interventions using various approaches were identified. Occupational therapists mainly manage intervention providers with multidisciplinary co-facilitator and parents’ involvement. Whilst session information varied, some evidence suggests that at least 30 min per session, minimum once per week up to twice per week, ranging from 10 to 23 sessions, may be sufficient. Intervention plans should be tailored to each child’s unique needs, taking into account the variety of available interventions. Collaboration among occupational therapists, parents, educators and health professionals in home programmes enhances self-care intervention outcomes. These results are set to inform future research and practice, paving the way for enhanced support and improved outcomes for children with developmental delays.

## Introduction

Early childhood years are crucial in terms of motor, social, emotional and cognitive development for productive performance in children’s development (1). All these skill areas are also essential for children to have the ability to perform self-care independently.

Self-care skills act as precursors for many school-related tasks and life skills (2). Self-care is part of the daily occupation which people undertake as individuals to occupy time and bring meaning and purpose to life (3). Typically, self-care refers to the skills of dressing and undressing, looking after oneself, using a toilet, taking food independently, bathing, washing and any other personal care (2). Self-care competence enables participation in life activities at school, home and the community (4). Developmental delay can render children unable to achieve typical developmental self-care tasks (5). Consequently, without the ability to complete self-care skills, children will have low self-esteem. A child’s self-esteem can suffer which leads to social isolation, lack of peer relationships and decline in their academic performance (2). In fact, children with difficulties such as the inability to independently use the toilet can cause them to be excluded from social and joint activities or recreation and public schools (5).

Developmental delay occurs when a child does not achieve developmental milestones compared to peers of the same age range (6). It can be detected through screening, parental concern or third parties such as teachers raising concerns. Children with developmental delay might have difficulty in one or more areas of children’s development, including self-care skills (6). Children with developmental delays can face problems related to lack of personal hygiene, especially in bathing and defecating (7) or difficulties when dealing with toileting, dressing and feeding (5). Some parents rated self-care skills as being of low concern in considering the range of therapy services (8). This situation may cause children to have less support or to be neglected in self-care skills development. Hence, children will develop a lack of self-confidence when they need assistance most of the time to perform their daily routine (9).

Self-care is an essential skill that children must perform in their daily routines. It is a part of the growing up process to achieve their development and maturation. This is because the preschool period of a child’s life is a precious time to achieve skills and to acquire evidence about the best strategies for fostering their development (10). Previously, studies were focusing self-care intervention on specific conditions such as autism, Down syndrome and haemophilia (11–15). However, there is little understanding of the evidence regarding intervention implementation among developmental delays. It is important to explore and improve self-care skills to ensure children have the best chance of developing adequate self-care skills performance.

Accordingly, parents, teachers, caregivers or therapists play roles in improving children’s self-care skills by identifying essential components related to existing self-care skill intervention. Therefore, the scoping review aims to explore and report on the type of intervention, frequency, timing, duration, method, intervention provider, intervention context and strategies/recommendations in intervention implementation for self-care skills among children with developmental delay. A scoping review of the existing research on self-care interventions for children with developmental delays can serve as the basis for refining clinical practise, guiding future research and ultimately improving the lives of these children and their families.

## Method

The researchers use five stages of planning of methodological framework based on the Joanna Briggs Institute (JBI) as described by Peters et al. (16), which has been refined, based on a framework developed by Arksey and O’Malley (17). The stages involve:

- Stage 1: Identifying the research questions
- Stage 2: Identifying relevant studies
- Stage 3: Selecting the relevant studies
- Stage 4: Charting the data
- Stage 5: Collating, summarising and reporting the results

### Stage 1: Identifying the Research Questions

The following research questions were considered in the study:

- What intervention exists to improve self-care skills in preschool children with developmental delays?
- How are these interventions provided in terms of frequency, duration, method, intervention provider and intervention context?
- What are the strategies or recommendations for the implementation of these interventions?

### Stage 2: Identifying Relevant Studies

To identify relevant studies, an extensive literature search was conducted using five databases from October 2021 to December 2023: Web of Science (W0S), Scopus, ASEAN Citation Index (ACI), CINAHL EBSCO and PubMed. The following keywords were used: ‘self-care’ OR ‘activity daily living’ OR ‘self-help’ AND ‘intervention’ OR ‘treatment’ OR ‘rehabilitation’ OR ‘Management’ AND ‘child*’ OR ‘preschool’ OR ‘kindergarten’ OR ‘early childhood’ OR ‘toddler’ AND ‘Occupational therapy’ AND ‘developmental delay’ with the use of MeSH terms, Boolean operators, parentheses and truncations whenever appropriate.

### Stage 3: Selecting the Relevant Studies

To be included in the review, the following eligibility criteria needed to be met: i) children with developmental delay, ii) studies published in English only, iii) children at the age of 3 months to 72 months, iv) quantitative and qualitative study design, v) interventions on self-care focused on improving self-care performance among children, vi) published between the years 2000 and 2023. Studies would be excluded if the study involved children with other health conditions such as autism and down syndrome and narrative, systematic or other review articles were excluded. Duplications of article were removed first before the screening process takes place. Each article was screened based on the eligibility criteria. The publications were assessed for eligibility by the first author, who examined the titles and abstracts of all the resources found. In accordance with the inclusion criteria and search phrases, a screening was conducted. Then, each researcher conducted a comprehensive screening of the chosen titles and abstracts to see whether their content was appropriate for inclusion in this review, such as whether it addressed the review’s questions. Following this, the researchers obtained the whole articles from the chosen abstracts, while abstracts that were not relevant were excluded. Finally, each whole article underwent a screening process to find pertinent information that addressed the review questions and to ascertain if it had achieved the objectives of the review. All disputes that arose throughout the screening phases among the reviewers were compared, and disagreements were deliberated until a consensus was achieved.

### Stage 4: Charting the Data

**Figure 1:**
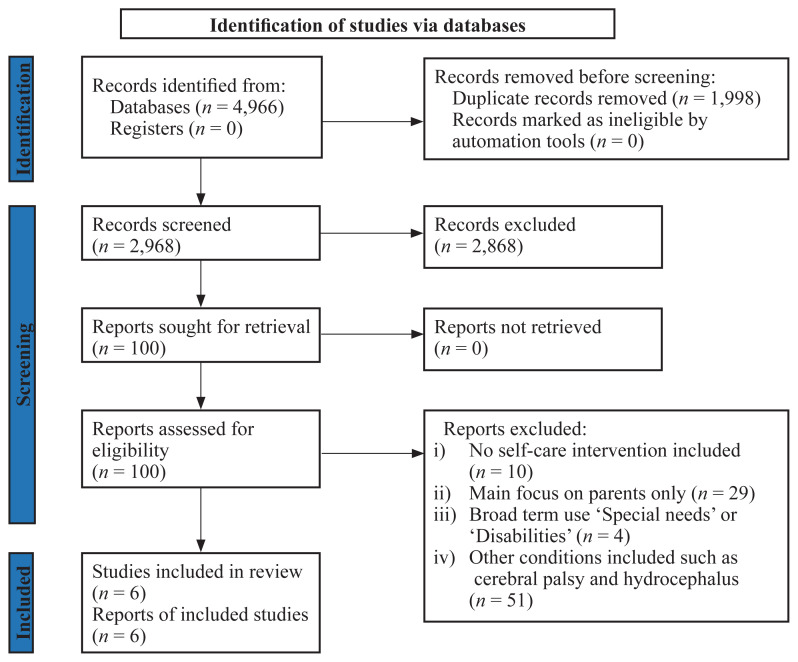
PRISMA-ScR flow diagram

Search results were saved and organised in Mendeley’s reference manager software database. In maintaining methodological rigour, the Preferred Reporting Items for Systematic Reviews and Meta-Analyses extension for scoping review guidelines (PRISMA-ScR) (18) was used to ensure that organisation, planning and reporting in this review was rigorous. See PRISMA flow diagram using software by Haddaway (19) (Figure 1).

Once the articles were selected, the next stage was charting the data, and data were recorded in a spreadsheet. Next, the reviewers analysed each paper independently and recorded details, including the author, year of publication, countries, study methodology, study aims, limitations/recommendations and study population. Others included types of intervention, duration, frequency, session, timing, intervention providers, range of age, intervention settings and strategies/recommendations. Then, the recorded pieces of evidence were ready for the next stage.

### Stage 5: Collating, Summarising and Reporting the Results

The final stage is collating, summarising and reporting evidence mapped by describing the results. The results of the extracted data were summarised and tabulated. As a result, several limitations of the studies were highlighted to address research gaps.

## Results

The search yielded a total of 4,966 papers (WoS = 627, Scopus = 190, ACI = 266, CINAHL EBSCO = 3,781 and PubMed = 102). After the removal of duplicates, 2,968 records remained. Following the screening of title and abstract, there remained 100 by independent reviewers; this was reduced to a final six selected papers. The full articles were excluded because of some reasons such as no self-care intervention included, intervention focus on parents instead of children and other reasons were not met the review’s objectives. The extracted results were classified under main conceptual categories based on the objective of this review (20), which consisted of: type of intervention, frequency, timing, duration, method, intervention context and intervention providers.

### Study Characteristic

A descriptive study design was used to report how specific skills activities such as motor skills help children in self-care skills performance (21) while pre-test-post-test, two-group control study design (22, 23), a single-subject study design (24, 25) and pilot randomised control trial reported on effectiveness of self-care intervention to children (26). Ages and demographics of the children varied greatly between studies. Most commonly, the age bracket of preschool children (3 years old–6 years old) and 3 months old–36 months old was included either as the primary focus or as part of a larger age inclusion from 36 months old to 72 months old. Selected studies in this review also came from both western (21, 22, 24) and Asian country (23, 25, 26), which provided the diversity of culture and different modes of intervention implementation (Table 1).

### Intervention Exists for Self-Care Skills among Children with Developmental Delay

**Figure 2:**
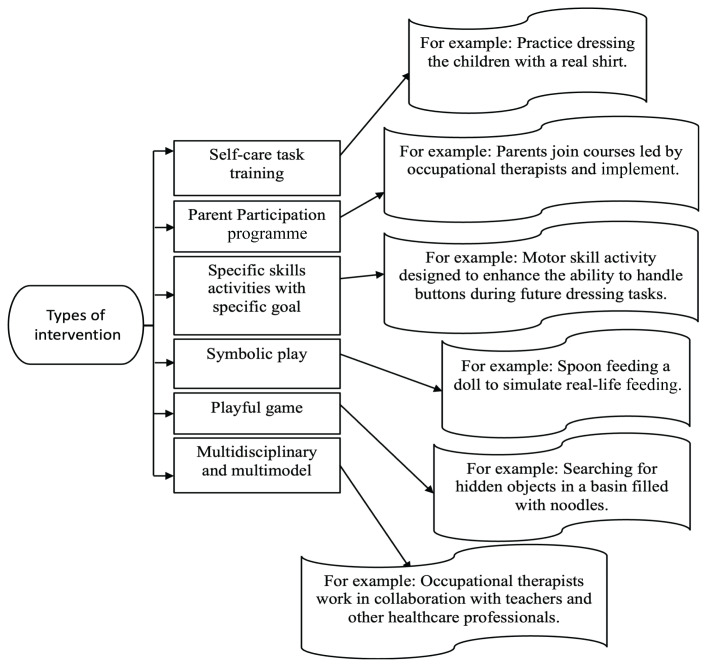
Intervention exists for self-care skills

The result shows (Figure 2) that various interventions to improve self-care skills performance are often used in combination to provide a holistic intervention for the child and his or her environment. One of the types of intervention is self-care task training (21, 24). The study systematically combines self-care task training with symbolic play and playful games (23, 24).

Symbolic play such as feeding dolls and playful games such as hide and seek were used to support and promote the children’s skills to improve their ability to be independent in self-care skills.

There were two studies using specific skills activities as an intervention which encompass elements including manual dexterity, gross motor (ball and balance) skills, graphomotor skills, cognitive skills, (22) sensory integration, in-hand manipulation, visual-motor integration and bilateral coordination (21). Child-specific skills interventions were done by applying multidisciplinary and multimodal (monitoring and collaborative consultation) early intervention programmes (22). In this framework, teachers and therapists jointly engage children in various activities, forming an integral part of the multidisciplinary and multimodal intervention strategy. Concurrently, therapists collaborate with parents to create a tailored home programme. This programme incorporates parents engaging in goal-focused activities or functional tasks at home with their children (23, 26) and these activities are then woven into their daily routines (23). This dual approach of professional-led and home-based activities has shown to be synergistic, leading to notable improvements in children’s skills, as well as their ability to perform and participate in self-care activities. Additionally, another study explored a combination of self-care task training, play, peer interaction and specific skill-focused activities (21) to foster self-care skills development.

### Frequency, Timing and Duration of Intervention

The duration of self-care interventions with children spanned 8 months in certain studies (21, 22), while others had durations of 2 months (25) and 3 months (23, 24, 26). Each method employed distinct intervention parameters, including the timing and frequency of sessions. The total number of sessions overall varied and ranged from 10 to 43 sessions. The frequency of the intervention sessions also differed, with some occurring once per week (21, 22, 24, 26) and other twice per week (25). There were home programme sessions designed for parents to incorporate activity sessions into their daily routines (23). The session duration varied from a minimum of 30 min (22, 25) and extending up to 90 min per session (21, 23, 24, 26).

### Method

A quasi-experimental study (25) was conducted by an occupational therapist who gave intervention with eight courses of occupational therapy and eight courses of parent participation for two groups of parents. There were two studies pilot randomised control trial (26) and pre-test-post-test experiment (23) implement home programme which parents need to do specific activities with children at home. Home programme was planned and guided by occupational therapist. The efficacy of the home programme is evaluated using two approaches: the home programme alone (23) or in combination with the in-clinic programme (26). Another study is a single-subject study design where the therapist gave intervention and made visits directly to the children (24). In a descriptive study by Case-Smith (21), the occupational therapy intervention was measured but not manipulated. The study described and recorded the intervention frequency and types of activities given to the children. Another study by Golos et al. (22) used a pre-test-post-test, two-group control study design. The group was divided into an intervention or a control group. The study included three phases: i) pre-testing and identification of the children at risk or with delays, ii) administration of the intervention programme and iii) post-testing.

### Intervention Provider

The main facilitators of treatment in these studies were certified and experienced occupational therapists (OTs) (21–26). One study involved only OTs (23, 24, 26), followed by OT combined with OT assistant (21). Another study recruited multidisciplinary providers such as teachers, speech therapist and educational counsellor (22). There were three studies which combined OTs and parents as collaborators in knowledge and providers of intervention (23, 25, 26). All studies highlighted positive outcomes even though there were different intervention providers involved except study done by Tang et al. (26).

### Intervention Context

Intervention programmes were typically carried out in the school environment (21, 22), hospital (25), home (23, 26) and public university (24). Based on intervention context, some studies stated the requirement that the therapist reschedule and negotiate for specific sessions due to parents and children missing treatment sessions.

### Strategies/Recommendations in Implementation of Interventions among Children with Developmental Delay

One of the studies, Joaquim et al. (24), revealed that children would develop and improve skills when they experienced activities in real tasks followed by simulation tasks and playful games. The children could learn self-care skills by experiencing those activities and reproducing them in symbolic games, such as with a doll. A systematic intervention plan is provided instead of ‘repetition’ of tasks without specific direction and goal on self-care skill. There was the ‘training’ in front of the mirror to assist in the experience and learning of the activity. Verbal cues and model demonstration were used for giving instructions, so they mimicked actions during the games and simulations of self-care skills. The study also emphasised the importance of a child being aware of his or her body in space to form the body schema, which is an elementary condition of the movements. Moreover, embedding the self-care training strategies into the family routines enhances self-care capabilities in a natural context, specifically home (25).

**Figure 3:**
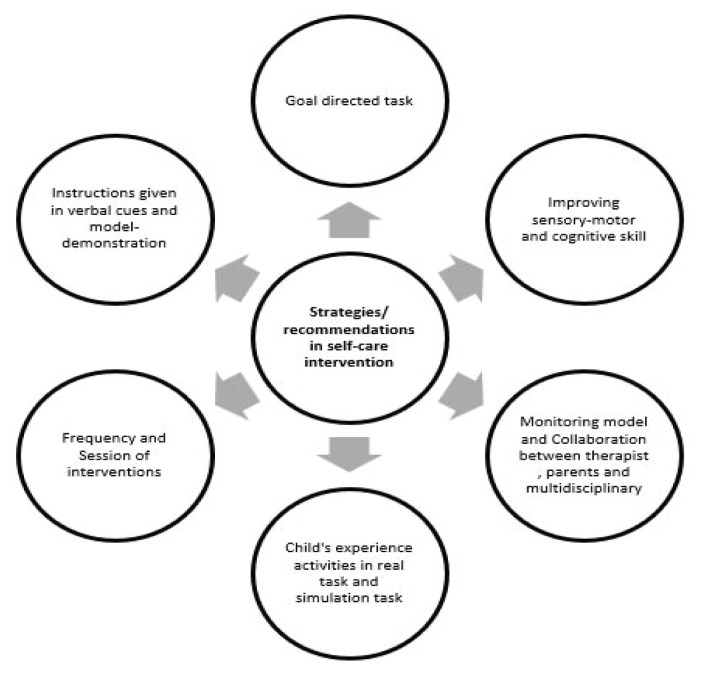
Strategies/recommendations in self-care interventions

The goal-directed task also positively impacted the child’s development. The study by Case-Smith (21) revealed the importance of motor accuracy and visual-motor skill to self-care function. Those skills benefit the child’s abilities in self-feeding, hygiene and dressing (21). It was mentioned that practising developmentally appropriate specific sensory-motor and cognitive skills may positively affect children’s ability to perform and participate in various daily living skills, including self-care skills (22). The intervention also emphasises precision and eye-hand coordination, which appears to have enhanced the child’s performance skills. The combined intervention models (multimodal and multidisciplinary), in which occupational therapists and educational staff work together and contribute their unique knowledge, may facilitate the ability to address children’s complex needs. Not only can children with developmental delays develop in all developmental fields, but a good communication bridge and better cooperation between parents and OTs can also be established, which will improve parents’ knowledge of and skills in parenting and provide information to support self-decision-making (25). Furthermore, home-based programmes, whether implemented independently (23) or in tandem with clinic-based interventions (26), are seen as effective strategies for increasing parental engagement. It is also significant to note, as pointed out by Case-Smith (21), that the impact on self-care skills is directly related to the frequency of sessions and the proportion of these sessions focused specifically on self-care objectives (Figure 3).

## Discussion and Implication

Although certain studies showed similar interventions, all six studies used different approaches and combinations to implement interventions for children. When looking at the type of interventions being conducted with clear strategies described, certain approaches may be more effective than others. Those with parents’ or families’ involvement demonstrated a faster improvement for children (23, 25). Specific task activities with clear self-care goals may benefit children with difficulties in the specific motor skills field (21, 22). Generally, no studies in this review mention any standardised protocol used, grading of each intervention, the level of resources regarding therapist and funding, materials, tools, procedure or details of whether the activities are carried out in a group or by individual children. Again, none of the interventions mentions specific strategies to maintain a child’s development scale after the intervention. It is essential to consider that information when implementing best practice, so that intervention providers can provide specific, congruent feedback while children practise each skill. Indeed, the intervention strategy was supported by children’s ability to maximise their learning through a high frequency of successful practice trials and positive congruent feedback (27). In all the reviewed studies, there was a reported enhancement in self-care skills, attributed to diverse research methodologies. However, an outlier was the study by Tang et al. (26), which noted no significant improvements in the self-care abilities of children. This deviation could be attributed to the smaller sample size used in their research and the loss of some participants during the follow-up period.

Looking at session frequency parameters, evidence suggests that an intervention programme should consist of a minimum of 30 min per session. The more frequently sessions occurred in a month, the sooner intervention results became evident in the children However, it is only an hypothesis based on six studies. There is also no specific length of time mentioned for each activity or task given as an intervention to the children, particularly task activities such as motor skills activities. However, each task given should have a specific duration to make it more systematic (27). Further, the longer time session might be needed if the intervention implementation occurs in the home context. Extra time is required to discuss, exchange information, and explain to parents during the intervention (28). Moreover, integrating intervention tasks into the daily routines is advised (23). Given the high caseloads in those settings, the time spent with each child on those goals is dictated more by the therapists’ caseloads and educational requirements in a specific system. Thus, any frequency and duration recommendations are based on a variety of factors that may limit the appropriateness of the recommended parameters. There could be so many variables that the that duration and frequency are no longer the most useful factors in making actual treatment decisions.

One study involved a small number of participants (24) by using a single subject study design that would lack solid evidence. This correlates with the previous study done by Handley et al. (29). The study’s power can be reduced if sample size considerations are not built in at the design phase (29). Nevertheless, quality assessment is not necessarily applicable to scoping studies in health research (30). Despite that, the systematic plan of intervention suggested in the study done by Joaquim et al. (24) could be a good example to practice. One pilot Randomised Controlled Trial (RCT) reported no significant enhancement in self-care skills through home programme interventions (26). Consequently, it is advisable to conduct additional RCTs or systematic reviews of existing RCTs focusing on self-care skill interventions for children with developmental delays. Such research would facilitate more informed choices concerning optimal intervention strategies, including the appropriate ratio of intervention providers to children per session and a standardised protocol for each intervention type.

OTs, followed by OT assistants, were found to be the most prominent providers of self-care intervention in line with the OT scope of the intervention (3). Although OT seems to remain a specialist area of care involving a range of specific treatment approaches, the search term used for this review, ‘occupational therapy,’ could contribute to the result revealed. So, this result should be further investigated in future reviews. Apart from this, other multidisciplinary participants, including teachers, speech therapists and an educational counsellor, were cooperative and helpful as co-facilitators. Parents are also involved with a therapist, depending on the service delivery model. Most of the studies in this review did not mention the exact service delivery models used, even though they might use similar models. Exceptionally, only one study highlighted multimodal methods, which involved monitoring and collaborative consultation (22) that seem to complement each other. They enable both improvements in the children’s skills and participation in self-care skills. It shows that having therapist, parents and multidisciplinary provider acting together could be beneficial to children’s intervention. It should also be emphasised that in home programme interventions, family members play the central role, while professionals provide training, supervision and evaluation. This creates a mode of intervention characterised by long-term collaboration and joint implementation. However, the level of resources involved with therapists, time and funding is important to consider when implementing best practice in the use of the service delivery models’ intervention (31). Further, the intervention provider, context and country requirements may be significant factors in delineating intervention providers for children who need to develop self-care skills. For instance, in the US school systems, multidisciplinary reinforcement of self-care skills in considered best practice. OTs often, although not always, establish the intervention plan and strategies to be used.

From this review, it seems that interventions were likely to occur in various contexts, including hospitals, schools, home, clinic, private centres and health schools at public universities. No interventions were reported in day care, kindergarten or social welfare settings. This might be due to the very few studies included in this review. Additionally, no studies mention whether intervention settings influenced the effectiveness of the intervention given. This correlates with one systematic review (32) that indicated no evidence that interventions delivered only in the hospital context are effective. By contrast, evidence illustrates that intervention that removes task and environmental barriers to children saw improvements in their self-care participation (33). The intervention contexts would be one of the crucial characteristics that should be considered when developing an intervention plan for children. Correspondingly, children displayed better performance with well-supported multifaceted aspects (34). Other influencing factors that significantly impact child development should also be considered such as socio-economic factors, accessibility, involvement of family, daily routine schedule and therapeutic resources.

The country where the studies were conducted should also be considered. Western countries and Asian countries have different socio-cultures, health systems and defined terms used. Developmental delay terms often used to specify a developmental delay may vary by country or practice area (35). Despite the developmental delay, patterns are often familial, including late walking and talking (36). Yet, for a diverse population with self-care skills difficulties, it is vital to conduct further studies in more countries to clearly define the term developmental delay and corresponding interventions.

The range of children’s ages should also be looked at, because each child’s ability to master self-care skills was different according to chronological age (37). For instance, according to the studies in self-care intervention that were reviewed, different age-related interventions were involved. The needs and demands of skills assistance and the goals of achieving skills enhancement are different according to the child’s age (38). Thus, intervention providers need to identify children’s ages and abilities required prior to the intervention plan.

This review had limited studies investigating developmental delay, as comorbidities to other diagnoses were omitted. However, this was done to ensure homogeneity of the population. Another reason for excluding comorbidities is that definition terms or categories of developmental delay differ from country to country (6). As well, this review only included reports in the English language, which potentially limited information on different cultural contexts. Nevertheless, Western and Asian studies that might support diverse populations regarding self-care intervention were included.

Five of the chosen studies also use different standardised assessments and one study use non-standardised tools. This limits conclusive comparisons between studies. This scoping review identifies the wide variability of results and findings of self-care intervention but is beyond the scope of a meta-synthesis or a meta-analysis.

## Conclusion

This scoping review has meticulously explored the landscape of interventions aimed at enhancing self-care skills in children with developmental delays. A diverse range of types and approaches are evident in early childhood interventions. Notably, the role of the OT is highlighted as a key element in these self-care interventions, with active involvement and collaboration with parents, educators, and other healthcare professionals, such as doctors, contributing to more rapid improvements. Additionally, home-based programmes are recognised as valuable supplements to therapy sessions, effectively complementing other intervention strategies. Moreover, this review not only verifies the presence of these interventions but also provides insights into their practical application and areas for potential enhancement. It underscores the necessity for additional research to detail intervention procedures, the grading of each intervention, the effectiveness in both group and individual settings, the delivery methods of intervention models and specific strategies or protocols to sustain children’s developmental progress post-intervention. Conclusively, this scoping review makes a significant contribution to the field by confirming the existence and detailing the operational characteristics of self-care interventions for preschool children with developmental delays. These findings are poised to guide future research and practice, ultimately leading to improved support and better outcomes for children with developmental delays.

## Figures and Tables

**Table 1:** Characteristic of studies from references included in this review

Authors	Year	Study design	Children’s age	Study setting	Sample size	Outcome measures	Interventions
Case-Smith (21)	2000	A descriptive	4 years old–6 years 0ld	United State of America	44	The motor accuracy test DTVP PDMS Draw-a-person test PEDI	Specific task activities
Golos et al. (22)	2011	Pre-test-post-test, two-group control study design	4 years old–6 years old	Israel	81	DTVP MABC MAP SPO	Multidisciplinary and multimodel early intervention programme
Dong et al. (23)	2023	Pre-test-post-test experimental	3 months old–24 months old	China	306	GMDS PSI/SF	A parent-implemented early intervention programme (PIEIP)
Joaquim et al. (24)	2018	A single-subject design	4 years old	Brazil	1	Operational portage inventory Portage guide to pre-school education	A systematised plan, with the ‘training’ of skills for activities of daily living, followed by playful and symbolic games
Lin et al. (25)	2018	Quasi experimental	10 months old–69 months old	Taiwan	60	CDIIT	Rehabilitation therapy and Parent participation programme
Tang et al. (26)	2011	A pilot randomised clinical trial	6 months old–48 months old	Taiwan	70	CDIIT PEDI	Institutional-based therapy programme (ITP) and home activity programmes (HAPs)

Notes: DTVP = Developmental Test of Visual Perception; PDMS = Peabody Developmental Motor Scales; PEDI = Paediatric Evaluation of Disability Inventory; MABC = Movement Assessment Battery for Children; MAP = Miller Assessment for Preschoolers; SPO = The Structured Preschool Observation; CDIIT = Comprehensive Developmental Inventory for Infants and Toddlers; GMDS = Griffiths Mental Development Scale; PSI/SF = Parenting Stress Index Short Form
